# CanVasc recommendations for the management of antineutrophil cytoplasm antibody (ANCA)-associated vasculitides – Executive summary

**DOI:** 10.1186/s40697-015-0078-1

**Published:** 2015-11-09

**Authors:** Lucy McGeoch, Marinka Twilt, Leilani Famorca, Volodko Bakowsky, Lillian Barra, Susan Benseler, David A. Cabral, Simon Carette, Gerald P. Cox, Navjot Dhindsa, Christine Dipchand, Aurore Fifi-Mah, Michele Goulet, Nader Khalidi, Majed M. Khraishi, Patrick Liang, Nataliya Milman, Christian A. Pineau, Heather Reich, Nooshin Samadi, Kam Shojania, Regina Taylor-Gjevre, Tanveer E. Towheed, Judith Trudeau, Michael Walsh, Elaine Yacyshyn, Christian Pagnoux, for the Canadian Vasculitis research network (CanVasc)

**Affiliations:** Department of Rheumatology, Mount Sinai Hospital, University of Toronto, Toronto, Ontario Canada; Current address: Centre for Rheumatic Diseases, Glasgow Royal Infirmary, Glasgow, UK; Division of Pediatric Rheumatology, Alberta Children’s Hospital, University of Calgary, Calgary, Alberta Canada; Division of Rheumatology, McMaster University, Hamilton and Langs Community Centre, Cambridge, Canada; Division of Rheumatology, QEII Health Sciences Centre and Dalhousie University, Halifax, Nova Scotia Canada; Division of Rheumatology, St. Joseph’s Health Care, London, Ontario Canada; Division of Pediatric Rheumatology, BC Children’s Hospital and University of British Columbia, Vancouver, British Columbia Canada; Division of Respirology, McMaster University, St. Joseph’s Healthcare, Hamilton, Ontario Canada; Division of Nephrology, Dalhousie University, Halifax, Nova Scotia; Section of Rheumatology, University of Manitoba, Arthritis Centre, Winnipeg, Manitoba Canada; University of Calgary, Calgary, Alberta Canada; Division of Internal Medicine, Hôpital Du Sacré-Coeur, Montréal, Québec Canada; Division of Rheumatology, McMaster University, Hamilton, Ontario Canada; Division of Rheumatology, Memorial University of Newfoundland, Nexus Clinical Research, St. Johns, Newfoundland Canada; Division of Rheumatology, Centre Hospitalier Universitaire de Sherbrooke, Québec, Canada; Division of Rheumatology, the Ottawa Hospital/University of Ottawa, Ottawa, Ontario Canada; Division of Rheumatology, Lupus and Vasculitis Clinic, McGill University, Montréal, Québec Canada; Gabor Zellerman Chair in Nephrology Research of Toronto, Division of Nephrology, University Health Network, Toronto, Ontario Canada; Division of Rheumatology and the Arthritis Program Research Group in Newmarket, Newmarket, Ontario Canada; Division of Rheumatology, Arthritis Research Canada, University of British Columbia, Vancouver, British Columbia Canada; Division of Rheumatology, University of Saskatchewan, Saskatoon, Saskatchewan Canada; Department of Medicine, Queen’s University, Kingston, Ontario Canada; Division of Rheumatology, CHAU Hôtel-Dieu de Lévis, Université Laval, Québec, Canada; Department of Medicine and Department of Clinical Epidemiology and Biostatistics, McMaster University, Hamilton, Ontario Canada; Division of Rheumatology, University of Alberta, Edmonton, Alberta

## Abstract

The Canadian Vasculitis research network (CanVasc) is composed of physicians from different medical specialties, including rheumatology and nephrology and researchers with expertise in vasculitis. One of its aims was to develop recommendations for the diagnosis and management of antineutrophil cytoplasm antibody (ANCA)-associated vasculitides in Canada. This executive summary features the 19 recommendations and 17 statements addressing general AAV diagnosis and management, developed by CanVasc group based on a synthesis of existing international guidelines, other published supporting evidence and expert consensus considering the Canadian healthcare context.

## Background

ANCA-associated vasculitides (AAV, including granulomatosis with polyangiitis [GPA], microscopic polyangiitis [MPA] and eosinophilic granulomatosis with polyangiitis [EGPA]) are potentially life-threatening vasculitides characterized by inflammation of small-sized blood vessels with resultant ischemic events, hemorrhagic events or both [1]. Their rarity and the heterogeneous nature of AAV mean that the management of individual patients can be extremely challenging and may vary markedly across different geographical regions and medical disciplines. Existing guidelines were initially developed prior to 2010, most have not yet been updated, and did not take into account the specificities of health care system delivery, access to services and drug treatments in Canada [2–11].

The Canadian Vasculitis research network (CanVasc) was created in November 2010 and its core committee includes physicians of different specialties, though primarily rheumatologists and nephrologists. One of the first major objectives of CanVasc was the development of recommendations for the management of patients with AAV within Canada, based on a synthesis of existing international guidelines, supporting evidence and expert consensus of a national Canadian AAV clinical and research network.

### Contexte

Les vascularites associées aux ANCA (les VAA, incluant la granulomatose avec polyangéite [GPA], la polyangéïte microscopique [MPA], et la granulomatose éosinophilique avec polyangéite [EGPA]) sont des vascularites caractérisées par une inflammation de la paroi des vaisseaux sanguins de petit calibre, aboutissant à des complications ischémiques ou hémorragiques, et qui mettent souvent en jeu le pronostic vital. Leur rareté, de même que leur présentations cliniques variées, rendent la prise en charge des patients extrêmement ardue, surtout si elle diffère selon les régions géographiques et les disciplines médicales. Les recommandations internationales de prise en charge des VAA existantes ont été développées avant 2010; la plupart n’ont pas encore été mises à jour et aucune ne tenait compte des spécificités de prestation des soins, d’accès aux services et aux divers traitements pharmacologiques dans le réseau de santé canadien.

Le groupe de recherche canadien sur les vascularites (CanVasc) a été créé en novembre 2010. Il est constitué de médecins de diverses spécialités, quoique surtout des rhumatologues et néphrologues. L’un des objectifs principaux de CanVasc était l’élaboration de recommandations de prise en charge des patients atteints de VAA au Canada, en se basant sur les recommandations internationales déjà existantes, les autres preuves scientifiques et publications dans le domaine des VAA, et un processus avec plusieurs étapes afin d’aboutir à un consensus.

## Methods

Prior to initiating the development of these recommendations, a national Needs Assessment Questionnaire was disseminated to identify the specific areas of need, possible knowledge gaps and outline key questions [12]. The international existing clinical practice guidelines and consensus statements on the management of AAV published in English or French between 2006 and May 2014 were then reviewed, in addition to Cochrane library and PubMed Medline searches for all therapeutic studies published after the 2009 European League against Rheumatism/European Vasculitis Society (EULAR/EUVAS) recommendations and May 2014. The first draft of these recommendations was developed by the core group of the CanVasc recommendation working group and included 37 recommendations, with the rationale behind each of them, the corresponding recommendations and guidance from other societies, when existing and the level of evidence categorized and graded according to the criteria previously endorsed by EULAR/EUVAS [2, 13]. This first draft was reviewed by all members of the CanVasc recommendation working group (using a modified Delphi method) with a phone conference held thereafter to reach consensus on all debated recommendations, especially those not agreed upon by >80 % of the reviewers. A revised version of recommendations was then developed and distributed again for review to the same working group and a broader spectrum of other reviewers, including members of several professional medical societies and specialists and the administrative bureau of the Canadian support group for vasculitis patients (Vasculitis Foundation Canada). The comments were gathered and discussed during a second teleconference with the members of the CanVasc recommendation working group to reach consensus on the final version of the document, which was endorsed by the Canadian Rheumatology Association (CRA) Guidelines Committee on March 21st, 2015.

## Results

The final document (the full version of the recommendations is available online at http://www.jrheum.org) includes 19 recommendations and 17 statements addressing general management strategies for AAV, including their diagnosis, treatments with glucocorticoids, traditional immunosuppressants and biologic agents, and follow up for rheumatologists, nephrologists, respirologists, general internists, general practitioners and all other health care professionals more occasionally involved in the management of patients with AAV in community and academic practice settings. Each therapeutic recommendation and statement is accompanied by supporting text, which reports on the expected health benefits, potential side effects and risks, and Canadian system factors that may influence their applicability. Therapeutic recommendations are presented with a level of evidence and strength (Table 1 of the executive summary). Statements are for non-therapeutic recommendations and working group consensus, for which there is no strong supporting evidence from controlled studies are not graded. For each recommendation and statement, we also present in the extended version (available online with the full version of the recommendations at http://www.jrheum.org) corresponding recommendations and guidance previously published on the same topic from other societies, when available.

**Table 1:** Summary table of the CanVasc recommendations and statements

Recommendation or statement	Text of the recommendation or statement	Evidence level/strength^a^
Diagnosis		
Statement 1	ANCA testing with ELISA and indirect immunofluorescence methods should be performed for diagnostic purposes in patients in whom there is clinical suspicion of a systemic small- and/or medium-sized vessel vasculitis.	
Statement 2	Tissue biopsy should be considered in cases of suspected AAV to confirm diagnosis.	
Classification of disease severity in AAV		
Statement 3	Patients with AAV should have the extent and severity of their disease categorized as ‘severe’ at the time of diagnosis and in case of subsequent relapse if they have life- or major organ-threatening manifestations in order to tailor therapy accordingly.	
The role of referral centres for vasculitis		
Statement 4	Patients with AAV, particularly those with challenging disease, should be managed at or in collaboration with, a referral centre for vasculitis.	
Remission induction for newly-diagnosed disease		
	*Remission induction in severe (organ/life-threatening disease) newly-diagnosed disease*	
Recommendation 1	We recommend remission induction therapy with a combination of high dose glucocorticoids and cyclophosphamide in patients with severe newly diagnosed GPA, MPA or EGPA.	*1B/A*
Recommendation 2	We recommend using high dose glucocorticoids with rituximab as 1st line remission induction therapy in patients with severe GPA or MPA in whom cyclophosphamide is contraindicated or in whom cyclophosphamide presents an unacceptable risk of infertility.	*1B/A*
Recommendation 3	Cyclophosphamide dose should be adjusted in patients >60 years of age and in those with renal impairment.	*1B/B*
Statement 5	Complete blood count (CBC) and serum creatinine level must be monitored in patients treated with cyclophosphamide. In patients with abnormal CBC results, temporary withholding of cyclophosphamide and subsequent dose adjustments may be necessary depending on the degree of leucopenia.	
Recommendation 4	We recommend that the remission induction therapy with cyclophosphamide, combined with glucocorticoids, lasts a minimum of 3 to a maximum of 6 months. Once remission is achieved, cyclophosphamide should be stopped and switched to a different maintenance therapy.	*1B/A*
Recommendation 5	We recommend that glucocorticoids should be given in adults at an initial dose of 1 mg/kg/day prednisone-equivalent for remission induction purposes. This may be preceded by pulsed methylprednisolone (0.5–1 g/day for 1–3 days) in patients with life threatening disease and/or major organ involvement.	*2A/B*
Recommendation 6	Prophylaxis against *Pneumocystis jiroveci* infection should be given to patients receiving cyclophosphamide or rituximab. This prophylaxis consists, in the absence of allergy, of trimethoprim/sulfamethoxazole compounds (800/160 mg 1 tablet 3 times per week or 400/80 mg daily).	*3/C*
Recommendation 7	There is insufficient evidence to support a recommendation that plasma exchange be used as first line therapy in any AAV patients. Plasma exchange may be a reasonable adjuvant therapy for patients who clinically deteriorate due to active vasculitis despite ongoing remission induction therapy with high-dose glucocorticoids and cyclophosphamide or rituximab.	*4/D*
	*Remission induction for limited or non-severe (non organ- and non life-threatening), newly-diagnosed disease*	
Recommendation 8	In patients with limited and/or non-severe GPA, which is non-life threatening and without any major organ involvement, remission induction regime with methotrexate in combination with glucocorticoids can be used.	*1B / A*
Recommendation 9	Patients with non-severe EGPA or non-severe MPA without renal involvement can be treated with glucocorticoids alone for remission induction. At present, there is no consensus on the use of any immunosuppressant agents in combination with glucocorticoids in patients with EGPA or MPA that are non-severe (including those with mononeuritis multiplex).	*2B/C*
Remission maintenance therapy		
Recommendation 10	In patients with severe AAV in remission after a combined cyclophosphamide-glucocorticoid-based induction treatment, maintenance therapy can be based on azathioprine or methotrexate, initially in combination with low-dose glucocorticoids. Leflunomide or mycophenolate may be alternative agents in patients not tolerating or with contra-indications to azathioprine and methotrexate.	*1B/B*
Recommendation 11	In patients with severe AAV in remission after a combined cyclophosphamide-glucocorticoid-based induction treatment, maintenance therapy with rituximab infusions is an alternative to azathioprine, especially for those patients with PR3-ANCA-positive GPA.	*1B/A*
Statement 6	To date there is no definitive evidence to guide decisions for maintenance therapy after remission induction with rituximab.	
Statement 7	Low dose glucocorticoids should be part of the initial remission maintenance therapy after remission is achieved; there is not enough evidence yet to support further recommendation on the optimal duration of low dose glucocorticoids.	
Recommendation 12	We recommend the use of azathioprine, methotrexate or their alternatives (as per Recommendation 10 and 11) for remission maintenance therapy to be continued for a minimum of 18 months after successful remission induction. There is not enough evidence yet to support further recommendation on the optimal duration of their use for maintenance.	*3/C*
Recommendation 13	The use of trimethoprim/sulfamethoxazole (800/160 mg twice daily) as remission maintenance therapy can be considered in GPA as an adjuvant to immunosuppressant or after the cessation of maintenance immunosuppressive treatment.	*3/C*
Recommendation 14	Topical therapies may be considered, in combination with the systemic therapy and in collaboration with ENT subspecialists, to alleviate the symptoms of upper airway and ENT disease.	*3/C*
Relapsing disease		
Recommendation 15	We recommend remission induction of a major organ- or life-threatening relapse with either cyclophosphamide or rituximab in conjunction with high dose glucocorticoids. In patients who already received cyclophosphamide for initial remission induction or a previous disease flare, we recommend using rituximab for remission re-induction.	*1B/A*
Recommendation 16	There is insufficient evidence to support a recommendation that plasma exchange be used as first line therapy in all patients with relapsing AAV with severe renal (GFR <50 ml/min) or pulmonary hemorrhage. Plasma exchange may be a reasonable adjuvant therapy for patients who clinically deteriorate due to active relapsing vasculitis despite ongoing remission induction therapy with high-dose glucocorticoids and cyclophosphamide or rituximab.	*4/D*
Recommendation 17	We recommend that relapses that are non-severe, i.e. non-life and non-organ threatening, be treated with an increase in glucocorticoid dose in addition to optimizing the patient’s concurrent immunosuppressant agent.	*3/C*
Refractory disease		
Recommendation 18	We recommend the use of rituximab, in combination with glucocorticoids, in patients with severe GPA or MPA who fail to respond to cyclophosphamide as remission induction therapy.	*3/C*
Statement 8	Patients with refractory disease should be managed in a referral centre for vasculitis in collaboration with subspecialists with experience in managing such patients.	
Statement 9	Patients with EGPA and persistent asthmatic symptoms, despite remission of their vasculitic manifestations, should be managed in collaboration with a physician subspecializing in asthma management.	
Additional and experimental therapies		
Statement 10	In patients in whom the aforementioned therapies are ineffective, contraindicated or poorly tolerated, consideration can be given to alternate, additional and/or experimental therapies in collaboration with a referral centre for vasculitis.	
Follow up of patients with AAV		
Statement 11	Patients with AAV should be followed regularly for many years with full clinical assessment and routine laboratory work to assess disease course and track for disease activity and disease- or treatment-related damage.	
Statement 12	All patients previously treated with cyclophosphamide should have a urinalysis every 3–6 months as a lifelong means of screening for cyclophosphamide-induced bladder toxicity. If micro- or macroscopic hematuria is present, in the absence of an alternate explanation, the patient should be referred for consideration of a cystoscopy.	
Statement 13	As part of their lifelong annual follow-up, cardiovascular risk factors (including smoking status, diabetes, hypercholesterolemia, hypertension and obesity) and risk for osteoporosis should be systematically assessed, with treatment as needed according to the current respective guidelines for each of these conditions.	
Special patient groups		
Statement 14	Women with AAV should not consider pregnancy earlier than 6 months after sustained remission of their disease has been achieved. Women with AAV planning pregnancy and those pregnant should be managed in close collaboration with an obstetrician with expertise in this field and/or in high-risk pregnancies.	
Statement 15	There are no pediatric specific management guidelines for pediatric AAV, and most knowledge in pediatric AAV is adapted from adult research. Management of children with AAV should be provided by pediatric physicians at an academic healthcare Centre, in collaboration with referral centres for vasculitis and/or centres with special interest in pediatric vasculitis.	
Statement 16	AAV in children should be classified at the time of diagnosis based on the childhood EULAR/PRINTO/PReS criteria in order to tailor therapy accordingly.	
Statement 17	Children with newly diagnosed AAV should be treated according to adult recommendations for induction of remission and then maintenance, with dose of medications adjusted for this specific population.	
Recommendation 19	In children, severe relapsing AAV or severe AAV refractory to the combination of cyclophosphamide and glucocorticoids (with major organ involvement or life-threatening manifestations) should be treated with rituximab, in combination with glucocorticoids.	*4/D*

^a^Statements are not related to specific treatments and were not be graded as there was no strong evidence or available studies to support them

## Discussion and conclusion

This document will serve as useful knowledge to support decision-making for any physician involved in the care of patients with AAV, including adults and children. Best clinical judgment must however always prevail when confronted with each specific patient scenario. New information from ongoing research may already have become available by the time the present document is published. Regular updates will thus be mandatory.
